# Efficient *N*-Glycosylation of the Heavy Chain Tailpiece Promotes the Formation of Plant-Produced Dimeric IgA

**DOI:** 10.3389/fchem.2020.00346

**Published:** 2020-04-22

**Authors:** Kathrin Göritzer, Iris Goet, Stella Duric, Daniel Maresch, Friedrich Altmann, Christian Obinger, Richard Strasser

**Affiliations:** ^1^Department of Applied Genetics and Cell Biology, Institute for Plant Biotechnology and Cell Biology, University of Natural Resources and Life Sciences, Vienna, Austria; ^2^Division of Biochemistry, Department of Chemistry, University of Natural Resources and Life Sciences, Vienna, Austria

**Keywords:** glyco-engineering, glycosylation, immunoglobulin, monoclonal antibody, recombinant glycoprotein

## Abstract

Production of monomeric IgA in mammalian cells and plant expression systems such as *Nicotiana benthamiana* is well-established and can be achieved by co-expression of the corresponding light and heavy chains. In contrast, the assembly of dimeric IgA requires the additional expression of the joining chain and remains challenging especially in plant-based systems. Here, we examined factors affecting the assembly and expression of HER2 binding dimeric IgA1 and IgA2m(2) variants transiently produced in *N. benthamiana*. While co-expression of the joining chain resulted in efficient formation of dimeric IgAs in HEK293F cells, a mixture of monomeric, dimeric and tetrameric variants was detected in plants. Mass-spectrometric analysis of site-specific glycosylation revealed that the *N*-glycan profile differed between monomeric and dimeric IgAs in the plant expression system. Co-expression of a single-subunit oligosaccharyltransferase from the protozoan *Leishmania major* in *N. benthamiana* increased the *N*-glycosylation occupancy at the C-terminal heavy chain tailpiece and changed the ratio of monomeric to dimeric IgAs. Our data demonstrate that *N*-glycosylation engineering is a suitable strategy to promote the formation of dimeric IgA variants in plants.

## Introduction

**Figure 1:**
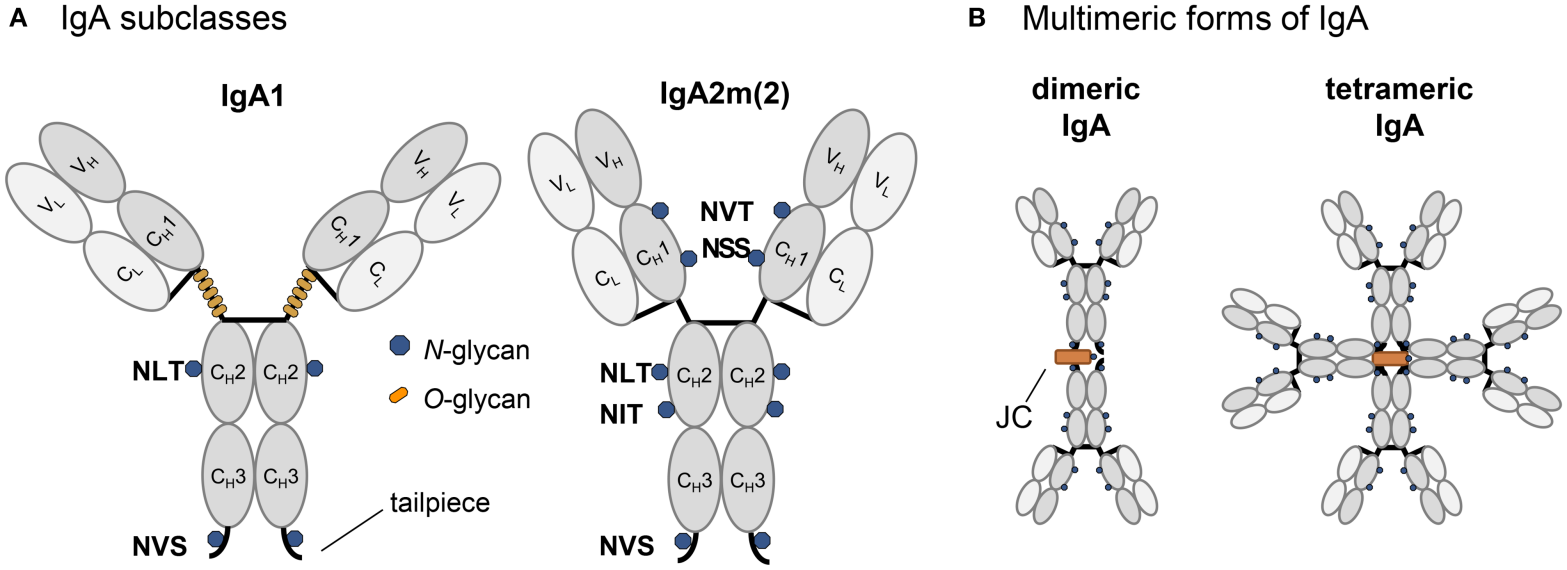
Schematic illustration of structure and glycosylation of IgA variants. **(A)** Representation of the IgA isotypes IgA1 and IgA2m(2) with the light chain colored in light gray and the heavy chain in dark gray, *N*-glycans are indicated by blue dots, *O*-glycans specific for the elongated hinge-region of IgA1 are indicated by orange dots. **(B)** Schematic presentation [only for IgA2m(2) shown] of multimeric states with the joining chain in orange.

Human immunoglobulin A (IgA) is the second most prevalent serum immunoglobulin after IgG and is the predominant antibody class in the external secretions of mucosal surfaces, where it serves as a first line of defense against invading pathogens. The human body expends a considerable amount of energy producing IgA variants thereby exceeding the daily production of all other immunoglobulin classes combined (Woof and Mestecky, 2005). This huge IgA demand highlights the importance of IgA in immune defense processes for which it is equipped with unique structural attributes of its heavy chain and its ability to form monomeric, dimeric and polymeric forms (Figure 1). While monomeric IgA is mainly found in serum and consists of two heavy chains (HC) and two light chains (LC), mucosal IgA is mainly dimeric whereby two IgA monomers are linked together by the incorporation of one joining chain (JC). In the dimeric IgA, the JC is covalently linked by disulfide bonds to the penultimate cysteine residue present in the C-terminal tailpiece of the IgA HC. The binding of the JC to the 18 amino acid long HC tailpiece is necessary for the formation of dimeric and polymeric IgA variants in the endoplasmic reticulum (ER) (Atkin et al., 1996; Yoo et al., 1999).

While recombinant IgGs are currently most widely used as therapeutic antibodies to combat infections or diseases, alternative antibody formats are gaining attention as potential biopharmaceuticals due to their specific structural properties and binding to different immune receptors (Loos et al., 2014; Brandsma et al., 2019; Montero-Morales et al., 2019). Polymeric antibody formats have a higher valency of antigen-binding sites which has several advantages compared to monomeric antibodies. Recombinant dimeric IgA against EGFR has been shown to be more effective in tumor cell killing than monomeric IgA or IgG1 due to the recruitment of a distinct repertoire of effector functions (Lohse et al., 2011). Moreover, dimeric IgA can bind to the polymeric immunoglobulin receptor (pIgR) and the pIgR-mediated transcytosis of dimeric IgA enables the access to therapeutic targets within the luminal side of mucosal tissues that are inefficiently targeted by current IgG therapeutics (Olsan et al., 2015). On the other hand, the formation of complexes between antiviral dimeric IgAs and viruses prevents the penetration of mucosal barriers (Ruprecht et al., 2019) and contributes to protection against mucosal virus challenge (Watkins et al., 2013). Together, these studies demonstrate the great potential of recombinant dimeric IgA as therapeutic agent. However, the production of dimeric IgA for clinical studies is technologically challenging due to the complex subunit assembly and extensive glycosylation (Vasilev et al., 2016).

Plants are attractive systems for the manufacturing of recombinant biopharmaceuticals including monoclonal antibodies (Stoger et al., 2014). Plant and mammalian cells share a common machinery for the biosynthesis and processing of *N*-glycans that is conserved up to the initiation of complex-type *N*-glycan formation in the *cis*/medial-Golgi (Strasser, 2016). On plant produced recombinant glycoproteins, complex and truncated *N*-glycans with β1,2-xylose and core α1,3-fucose are frequently found. These non-human residues are potentially immunogenic and numerous strategies have been employed to prevent their attachment to *N*-glycans (Montero-Morales and Steinkellner, 2018). These efforts resulted in the formation of human-like *N*-glycan structures on recombinant glycoproteins including different immunoglobulin classes (Strasser et al., 2008; Loos et al., 2014; Göritzer et al., 2017; Montero-Morales et al., 2019) and showed that plants tolerate extensive engineering of their glycan structures. Different plant expression systems have been used to produce monomeric, dimeric or secretory IgA variants (Ma et al., 1995; Karnoup et al., 2005; Juarez et al., 2013; Virdi et al., 2013; Paul et al., 2014; Westerhof et al., 2014, 2015; Dicker et al., 2016; Göritzer et al., 2019).

In a previous study, we have transiently expressed monomeric HER2 binding IgA1 and two IgA2 allotypes in leaves of *N. benthamiana* to examine the site-specific *N*-glycosylation (Göritzer et al., 2017). While we found no differences in glycosylation efficiency on most of the *N*-glycosylation sites compared to human cell-derived IgAs, the *N*-glycosylation site present in the tailpiece of all plant-produced IgAs was only 40–60% glycosylated. Here, we investigated how efficient dimeric HER2 binding IgAs are produced in the transient *N. benthamiana* expression system and whether the *N*-glycosylation in the tailpiece plays a role in the assembly of multimeric IgA.

## Materials and Methods

### Construct Design and Cloning

All constructs used for the expression of monomeric human HER2 binding IgA1 and IgA2m(2) isotypes in *N. benthamiana* and HEK293F cells have been described in detail recently (Göritzer et al., 2017, 2019). The codon optimized gene for expression of the joining chain (JC) (AK312014.1) in either *N. benthamiana* or HEK293F cells was synthesized by GeneArt (Thermo Fisher Scientific, USA) and was flanked with the signal peptide from barley alpha-amylase (AAA98615) and the restriction sites *Xho*I/*Age*I (for expression in *N. benthamiana*) or the signal peptide “MELGLSWIFLLAILKGVQC” and the restriction sites *Bam*HI/*Sal*I (for expression in HEK293F). The synthesized DNA fragments were cloned into the binary vector pEAQ-HT (Sainsbury et al., 2009) and the mammalian expression vector gWIZ (Genlantis, San Diego, CA) for expression of dimeric IgA in *N. benthamiana* and HEK293F, respectively, as described previously (Göritzer et al., 2017, 2019).

For the expression of the marginal zone B and B1-cell-specific protein (MZB1) (Q8WU39) in *N. benthamiana* the codon-optimized MZB1 coding sequence was synthesized by GeneArt. A construct for the expression of a tagged MZB1 (mRFP-MZB1) was obtained by amplification with the primers “TATATCTAGAGATAGGGCTCCTCTTACTGCTA”/“TATAGGATCCTCAAAGTTCCTCTCTGGTAGC,” digestion with the restriction enzymes *Xba*I/*Bam*HI, and cloning into *Xba*I/*Bam*HI digested p117 (Shin et al., 2017). For the expression of BiP2, the coding sequence was amplified from *A. thaliana* cDNA using the primers “TACTAGTATGGCTCGCTCGTTTGGAGCAAACAGCACT”/”TACTAGTCTAGAGCTCATCGTGAGACTCATCT” and subcloned using a Zero Blunt TOPO PCR Cloning Kit (Thermo Fisher Scientific, USA). The cloned fragment was excised by *Spe*I digestion and ligated into *Xba*I digested expression vector p42. Vector p42 is derived from pPT2M (Strasser et al., 2005) and carries the *A. thaliana* ubiquitin 10 promoter instead of the CaMV35S promoter and the sequence for the attachment of a 3x HA-tag plus the HDEL peptide at the C-terminus of the expressed protein. The CRT2 coding sequence was amplified from *A. thaliana* cDNA using the primers “TATATCTAGAATGGCGAAAATGATTCCTAGCC”/”TATAGGATCCAGCGGTGGCGTCTTTCTCAGAGG.” The PCR product was *Xba*I/*Bam*HI digested and cloned into the expression vector p59 (Schoberer et al., 2019) to express CRT2 fused to mRFP-HDEL. CNX1 was amplified from *A. thaliana* cDNA using “TATATCTAGAGACGATCAAACGGTTCTGTATG”/”TATAGGATCCCTAATTATCACGTCTCGGTTGCC,” *Xba*I/*Bam*HI digested and cloned into expression vector p110 (same vector as p117 but with a kanamycin resistance gene for selection in plants) to express an mRFP-CNX1 variant. For endoplasmic reticulum resident protein 44 (ERp44) expression, a codon-optimized coding sequence of human ERp44 (CAC87611) including the sequence coding for the signal peptide from barley alpha-amylase was synthesized by GeneArt and cloned into the *Xba*I/*Bam*HI sites of pPT2M. The expression construct of the single-subunit oligosaccharyltransferase from *Leishmania major* (LmSTT3D) has been described previously (Castilho et al., 2018).

### Expression and Purification of Dimeric IgA

For the expression of different recombinant monomeric and dimeric IgA isotypes in 5 to 6 weeks old *N. benthamiana* ΔXT/FT plants, syringe-mediated agro-infiltration was used (Strasser et al., 2008; Göritzer et al., 2017). To obtain dimeric IgA variants, the κ-LC and respective α-HC were co-infiltrated with the JC with an OD_600_ of 0.1 or 0.2. Chaperones were co-infiltrated at an OD_600_ of 0.05. To increase the *N*-glycosylation occupancy, IgAs were co-infiltrated with LmSTT3D at an OD_600_ of 0.1. After 4 days, infiltrated leaf material was harvested and the clarified crude extract was prepared for IgA purification as described previously (Göritzer et al., 2017). For the transient expression of monomeric and dimeric IgA isotypes in HEK293F cells, cultures were transfected with the κ-LC, the different α-HCs and JC constructs in a 1:1:0 and 1:1:0.5 ratio of μg DNA, respectively, as described (Göritzer et al., 2019). Finally, IgA from clarified *N. benthamiana* ΔXT/FT leaf extract and supernatant of HEK293F cells was purified with IgA CaptureSelect affinity resin (Thermo Fisher Scientific, US), followed by a size-exclusion chromatography step (Göritzer et al., 2017).

### SDS-PAGE

For reducing or non-reducing SDS-PAGE 2.5 μg of purified protein were loaded on a 4–15% Mini-PROTEAN^®^ TGX™ gel (Bio-Rad laboratories, USA) and detected by Coomassie Brilliant Blue staining.

### Size-Exclusion Chromatography Coupled to Multi-Angle Light Scattering (SE-HPLC-MALS)

To investigate the oligomeric state, conformational integrity and molecular weight of purified IgAs, high performance-liquid-chromatography (HPLC) coupled to a size-exclusion chromatography column (Superdex 200 10/300 GL column, GE Healthcare, USA) combined with multi-angle light scattering were carried out as described previously (Göritzer et al., 2017). HPLC (Shimadzu prominence LC20) was equipped with MALS (WYATT Heleos Dawn8+ QELS; software ASTRA6), refractive index detector (RID-10A, Shimadzu) and a diode array detector (SPD-M20A, Shimadzu). Ratios of monomeric, dimeric and polymeric IgA were determined by peak-integration using LabSolutions Data Analysis (Shimadzu) software.

### ELISA

Purified human HER2 (residues 1–631) was provided by Elisabeth Lobner (University of Natural Resources and Life Sciences, Vienna). For antigen-binding experiments of monomeric, dimeric and polymeric IgA variants ELISA was performed as described recently (Göritzer et al., 2017).

### Surface Plasmon Resonance (SPR) Spectroscopy

Binding experiments of monomeric and dimeric IgA variants to FcαRI were performed with surface plasmon resonance spectroscopy using a Biacore T200 (GE Healthcare Life Sciences, Sweden). Recombinant soluble FcαRI was available from a previous study (Göritzer et al., 2019). All measurements were conducted with a Protein L sensor chip (GE Healthcare Life Sciences, Sweden) as described recently (Göritzer et al., 2019). Binding affinities (*K*_D_) were calculated with the Biacore T2 Evaluation software using a 1:1 binding model. All experiments were repeated as three independent kinetic runs.

### *N*-Glycan Analysis

A total of 20 μg purified protein was reduced, S-alkylated and digested with trypsin (Promega, USA). Glycopeptides were then analyzed by capillary reversed-phase chromatography and electron-spray mass spectrometry using a Bruker Maxis 4G Q-TOF instrument (Göritzer et al., 2017). Site-specific glycosylation occupancy was calculated using the ratio of deamidated to unmodified peptide determined upon *N*-glycan release with PNGase A (Europa Bioproducts).

## Results

### Dimeric IgA Variants Are Less Efficiently Formed in *N. benthamiana*

**Figure 2:**
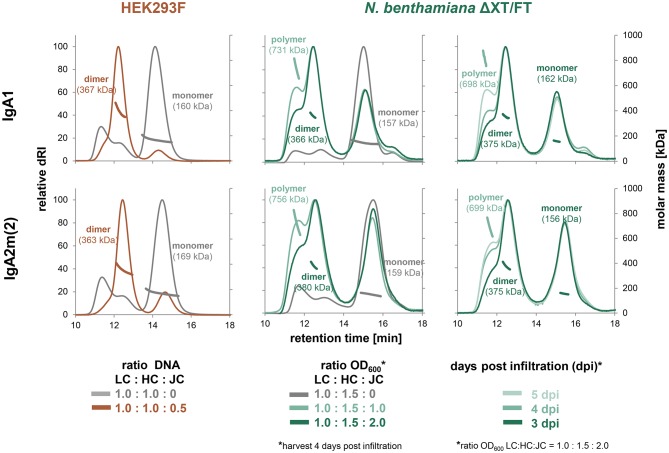
Factors contributing to dimer and oligomer formation of IgA. Overlays of normalized analytical size-exclusion HPLC-MALS chromatograms of affinity-purified IgA from small-scale transient transfections in HEK293F cells and small-scale infiltrations in *N. benthamiana* ΔXT/FT plants performed with varying ratios of light chain (LC), heavy chain (HC) and joining chain (JC) DNA in μg (HEK293F) or infiltration with different OD_600_ (*N. benthamiana*), respectively. Values were normalized based on the highest signal of each chromatogram.

To obtain a better understanding of dimeric IgA assembly, HER2 binding monomeric and dimeric IgA1 and IgA2m(2) (Figure 1) were transiently expressed in HEK293F cells and glyco-engineered *N. benthamiana* ΔXT/FT plants. Therefore, the κ-LC and respective α-HC were co-expressed in the presence and absence of the JC, followed by affinity purification and analysis of the assembly using SE-HPLC coupled to multi-angle light scattering (MALS). This allowed the determination of the molecular mass of the proteins in solution and quantification of the relative amounts of the different species using peak integration. Size-exclusion chromatograms showed that relatively pure monomers of IgA1 and IgA2m(2) with a mass of ~160 kDa are produced in the absence of the JC. In both expression systems, only small amounts of IgA with a molecular weight >160 kDa could be observed (Figure 2). Co-transfection of the JC resulted in almost complete formation of dimeric IgAs with a molecular mass of around 360 kDa in HEK293F cells. By contrast, a mixture of monomeric, dimeric and polymeric species was observed in plants. Thereby, the assembly of dimeric IgA1 appeared to be more efficient than the assembly of dimeric IgA2m(2). The formation of polymeric IgA, however, was dependent on the relative amount of JC co-transfected with the κ-LC and α-HC and the harvesting time after infiltration. Increasing ratios of JC to κ-LC and α-HC in the infiltration mix resulted in a decreased percentage of polymeric IgA. Furthermore, a later harvesting point yielded higher amounts of polymeric IgA (Figure 2 and Figure S1).

### Monomeric and Dimeric/Tetrameric IgA Display Similar Antigen and Receptor Binding Affinities

**Figure 3:**
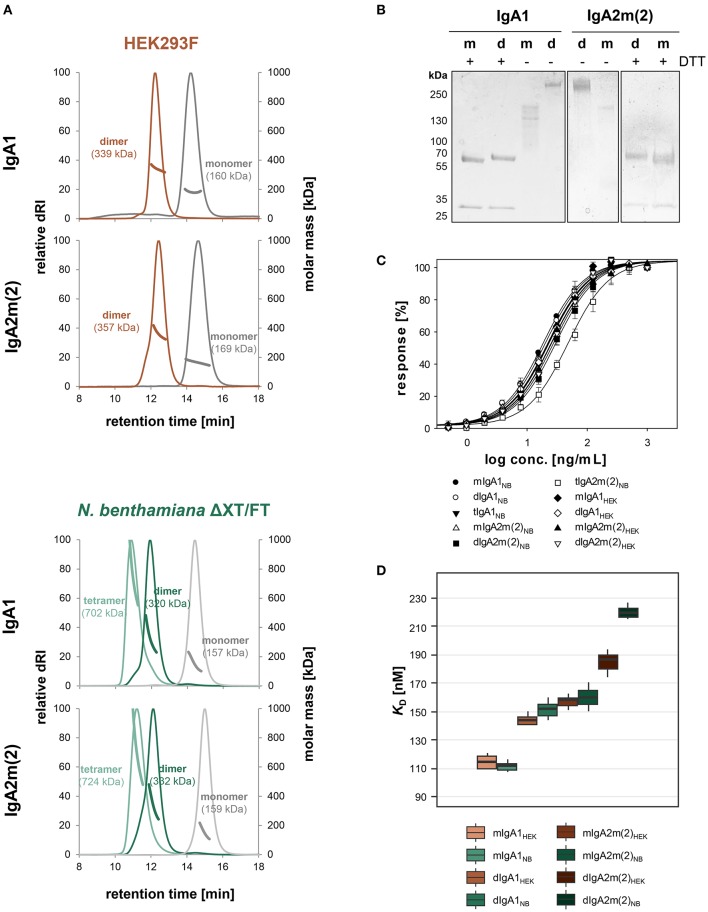
Biophysical and functional characterization of recombinant monomeric, dimeric and tetrameric IgA. **(A)** Overlay of normalized SE-HPLC-MALS chromatograms of affinity and gel-filtration purified IgA1 and IgA2m(2) monomers, dimers and tetramers produced in HEK293F cells and *N. benthamiana* ΔXT/FT plants. **(B)** SDS-PAGE under reducing (+DTT) and non-reducing conditions of purified monomeric (m) and dimeric (d) IgA1 and IgA2m(2) produced in *N. benthamiana* ΔXT/FT plants followed by Coomassie Brilliant Blue staining. **(C)** Binding of the IgA variants to the antigen HER2. The EC_50_ vales were determined as the mean ± standard deviation from three independent measurements. “m” monomeric, “d” dimeric, “t” tetrameric IgA. **(D)** Binding affinities of IgA1 and IgA2m(2) monomers and dimers to FcαRI. *K*_D_ values were obtained by SPR spectroscopy in single-cycle kinetic experiments from three independent measurements. Error bars represent standard deviation.

To determine the biophysical and biochemical properties of different IgA forms we up-scaled the production in both expression systems, followed by separate isolation of monomeric, dimeric and tetrameric species after affinity chromatography using preparative size-exclusion chromatography. The analytical profiles of all purified variants from the two different expression systems gave narrow single and monodisperse peaks (Figure 3A). The masses of these peaks were confirmed by MALS and represent fully assembled monomeric, dimeric and tetrameric IgA (present in plant-derived variants), with masses corresponding well to their theoretical masses. The SDS-PAGE of plant-produced monomeric and dimeric IgA1 and IgA2m(2) under reducing conditions confirmed the presence of the κ-LC and α-HC without the presence of degradation products (Figure 3B). Interestingly, under reducing conditions a small shift in the migration behavior of the α-HC of dimeric compared to monomeric IgA1 and IgA2m(2) was observed, which could arise from the presence of differentially processed *N*-glycans on monomeric and dimeric IgA. The JC (15 kDa) of dimeric variants could not be detected on the gel likely due to its low abundance. Under non-reducing conditions purified monomeric and dimeric IgA1 and IgA2m(2) variants showed a predominant band in the range of the expected molecular mass of 160 kDa for each monomeric variant and at 320 kDa for each dimeric variant, representing the assembled forms.

Next, we investigated the functionality of all purified IgAs in terms of binding to the antigen HER2. ELISA experiments were performed and the half-maximal effective concentrations (EC_50_) were determined (Figure 3C). Thereby it could be shown that the antigen binding behavior of monomeric and dimeric IgA1 and IgA2m(2) from plant and mammalian hosts is essentially the same and only tetrameric IgA2m(2) expressed in *N. benthamiana* showed slightly decreased binding to the antigen.

The monomeric and dimeric variants of IgA1 and IgA2m(2) were further tested for binding to the Fcα-receptor (FcαRI) using surface plasmon resonance (SPR) spectroscopy (Figure 3D). Therefore, the different IgA variants were immobilized on a Protein L chip in an oriented manner with the Fc-domain pointing toward the solution. Five increasing concentrations of the FcαRI were injected in single-cycle kinetic experiments. The obtained response units suggested a 1:1 binding stoichiometry for monomeric and dimeric IgA variants to the receptor and curves were fitted accordingly. The *K*_D_ values around 110 nM and 170 nM obtained for the HEK293F- and plant-derived monomeric IgA1 and IgA2m(2) variants, respectively, corresponded to the previously reported values using this set-up (Göritzer et al., 2019). A rapid association and dissociation rate was characteristic for the interaction of FcαRI with all IgA variants, whereas a decreased association rate for dimeric IgAs could be observed resulting in a slightly reduced binding affinity compared to the monomeric IgA variants (Figure 3D and Table S1).

### The *N*-Glycans of Plant-Derived Dimeric IgAs Are Different From Monomeric Variants

**Figure 4:**
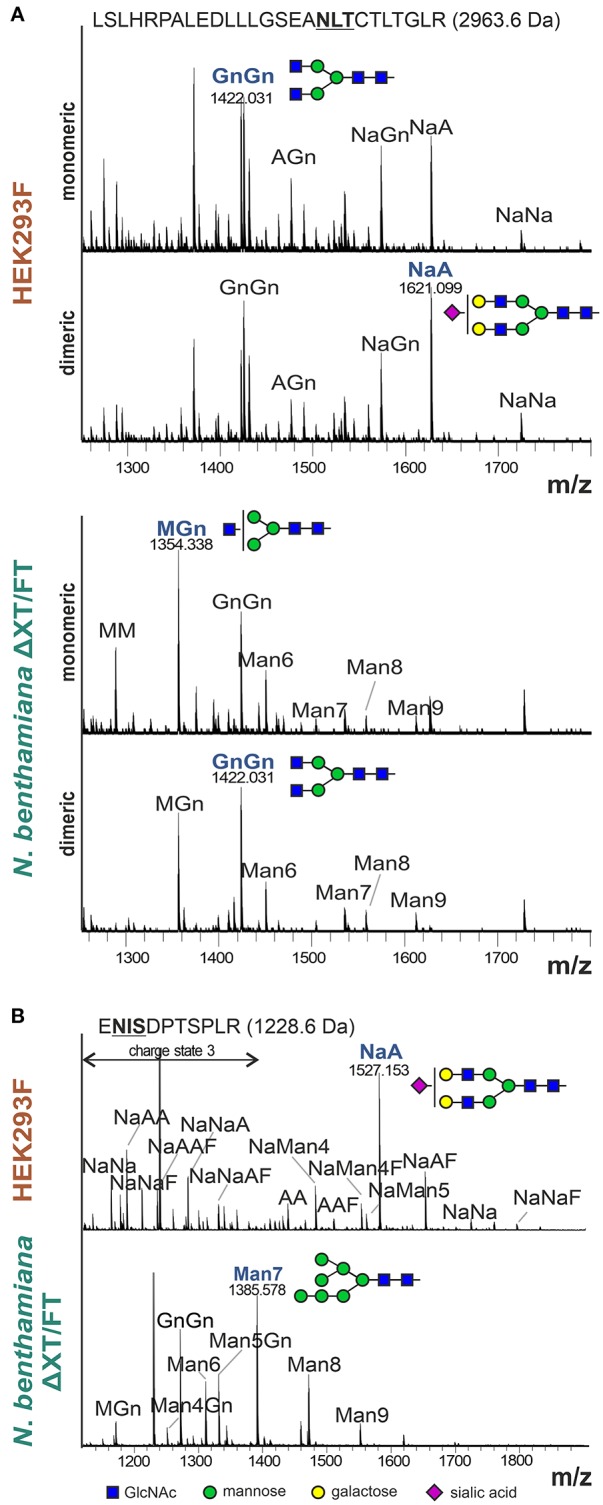
*N*-glycan analysis of the α-HC and JC from purified monomeric and dimeric IgA1. **(A)** Representative MS-spectra ([M+3H]^3+^) of the tryptic glycopeptide containing the CH2 resident NLT glycosylation site of the α-HC of HEK293F- and plant-produced IgA1. **(B)** Representative MS-spectra ([M+2H]^2+^ and [M+3H]^3+^) of the tryptic glycopeptide containing the single NIS glycosylation site of the HEK293F- and plant-produced dimeric IgA1 joining chain. *N*-glycans are abbreviated according to the ProGlycAn system (www.proglycan.com). The most abundant glycoform is highlighted in blue and illustrated with cartoons.

In the reducing SDS-PAGE of monomeric IgA1 and IgA2m(2) produced in *N. benthamiana* the shift in the mobility between monomeric/dimeric forms may arise from differential glycosylation. There are two and five *N*-glycosylation sites in IgA1 and IgA2m(2), respectively (Figure 1). In addition, IgA1 has up to 6 *O*-glycosylation sites in the proline-rich hinge region (Royle et al., 2003; Göritzer et al., 2017). To assess the *N*-glycosylation status of purified monomeric and dimeric IgA1 and IgA2m(2) produced in *N. benthamiana* and HEK293F cells, the purified proteins were digested with trypsin and analyzed by LC-ESI-MS for site-specific *N*-glycosylation and the presence of modifications within the IgA1 hinge region. The *N*-glycans found on plant-produced monomeric IgA1 showed biantennary complex-type structures like GlcNAc_1_Man_3_GlcNAc_2_ (MGn/GnM), GlcNAc_2_Man_3_GlcNAc_2_ (GnGn) and the paucimannosidic Man_3_GlcNAc_2_ (MM) as major glycoforms. In addition, small amounts of oligomannosidic *N*-glycans were detected on some of the sites (Figure 4A and Figures S2, S3). HEK293F-produced monomeric IgA variants have a more diverse profile with different amounts of branched or sialylated complex *N*-glycans. While most of the complex *N*-glycans are fucosylated Figures S2, S3), the conserved NLT site (Figure 1) lacks core fucose on monomeric as well as on the dimeric HEK293F-derived variants (Figure 4A). This finding is in accordance with the site-specific differences occurring on the monomeric IgA isotypes that have been described (Göritzer et al., 2017). Generally, the *N*-glycosylation profile of HEK293F-produced dimeric IgAs was similar to monomeric IgAs. For plant-produced dimeric IgAs a clear shift of paucimannosidic (MM structures) toward fully processed complex *N*-glycans (GnGn structures) was observed for the NLT site of dimeric IgA1 (Figure 4A), as well as for the NVT, NSS, and NIT sites of dimeric IgA2m(2) (Figure S3). By contrast, *O*-glycans present in the IgA1 hinge region appeared similar in monomeric and dimeric variants (Figure S4).

Furthermore, we were able to detect the single glycopeptide corresponding to the JC of HEK293F- and plant-produced dimeric IgA variants (Figure 4B and Figure S5). The *N*-glycan profile of the single site in the JC of HEK293F-produced IgA1 and IgA2m(2) showed a high heterogeneity with high levels of branching and incomplete sialylation and some peaks corresponding to core-fucosylated and hybrid *N*-glycans. In plant-produced JC derived from purified dimeric IgA1 and IgA2m(2) variants, the *N*-glycans were more homogenous with the GnGn-type complex *N*-glycan and oligomannosidic structures as major glycoforms and low levels of unglycosylated JC. The presence of oligomannosidic *N*-glycans suggests incomplete processing of the JC *N*-glycans in the Golgi of plants.

### Co-expression of ER-Resident Proteins Increased the Overall Yield, but Did Not Improve Dimeric IgA Formation in *N. benthamiana*

**Figure 5:**
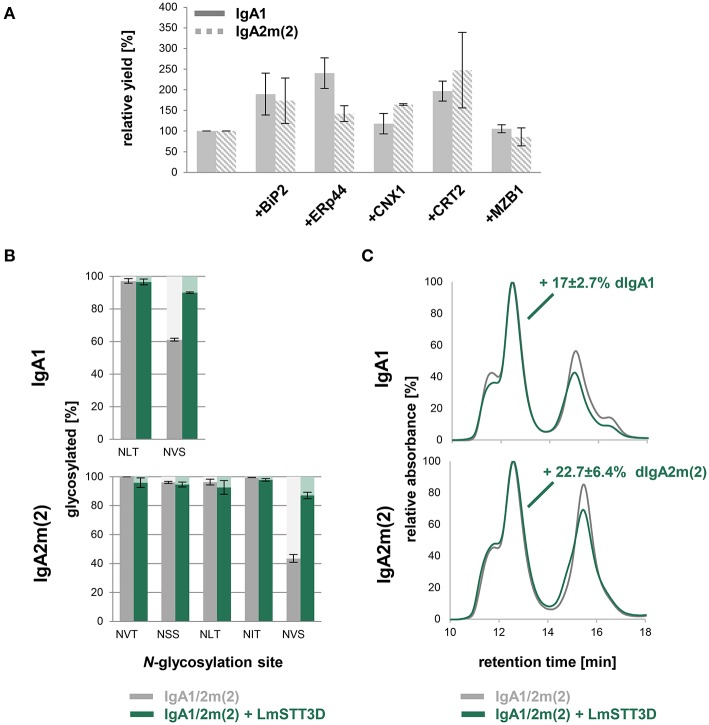
Optimization of dimeric IgA assembly in plants. **(A)** Relative yields of purified dimeric IgA1 and IgA2m(2) co-infiltrated with different ER-resident proteins in *N. benthamiana* ΔXT/FT. The bars indicate the average relative yield and error bars indicate the standard error (*n* = 4). **(B)** *N*-glycosylation site occupancy of plant-produced dimeric IgA1 and IgA2m(2) either infiltrated alone or co-infiltrated with LmSTT3D. Each value is the mean ± standard deviation from four independent experiments. **(C)** Overlay of normalized SE-HPLC chromatograms of affinity-purified dimeric IgA1 and IgA2m(2) with or without co-infiltrated LmSTT3D. The increase of dimeric IgA compared to monomeric and polymeric forms is the mean ± standard deviation from four independent experiments.

Despite the JC co-expression, plants were less efficient in assembly of dimeric IgAs compared to HEK293F cells with still large amounts of monomeric IgA present. Therefore, we investigated whether this limitation can be overcome by co-expression of different ER-resident proteins including the chaperone BiP, which is known to play a role for the antibody assembly (Haas and Wabl, 1983), the protein disulfide isomerase ERp44 and the lectins calnexin (CNX) and calreticulin (CRT). Human ERp44 binds to the tailpiece of the IgM HC, which is quite similar to the IgA tailpiece and promotes IgM polymerization in the ER of mammalian cells (Cortini and Sitia, 2010). The lectins CNX and CRT bind to immature *N*-glycans and promote the folding of glycosylated proteins (Hammond et al., 1994). In addition, we co-expressed the human marginal zone B and B-1 cell-specific protein MZB1, which was recently shown to promote JC binding and dimeric IgA assembly in mammalian cells (Xiong et al., 2019). The κ-LC, α-HC, and JC were co-infiltrated with either Arabidopsis BiP2, CRT2-mRFP, mRFP-CNX1, human ERp44, or mRFP-MZB1. None of these ER-resident proteins increased the relative amount of dimeric to monomeric IgA (Figure S6). However, upon using BiP2, CRT2, CNX1, and ERp44 higher yields could be achieved with up to 2-fold increase of purified IgA per gram fresh weight (Figure 5A).

### Increased *N*-Glycosylation of the Tailpiece Promotes Dimeric IgA Formation in *N. benthamiana*

Previous studies in mammals have indicated an important role of the tailpiece *N*-glycan for JC incorporation (Atkin et al., 1996; Sørensen et al., 2000). In plant-produced IgAs, the tailpiece is incompletely glycosylated which might contribute to inefficient dimeric IgA formation (Westerhof et al., 2015; Göritzer et al., 2017; Castilho et al., 2018). Underglycosylation was even more pronounced in the IgA2m(2) isotype which also exhibited higher amounts of non-assembled monomeric IgA when co-infiltrated with the JC. In a previous study we have shown that it is possible to overcome the reduced glycosylation efficiency by co-expression of LmSTT3D, a single subunit oligosaccharyltransferase from the protozoan *Leishmania major* (Castilho et al., 2018). Using this approach the occupancy of the tailpiece *N*-glycosylation site of dimeric IgA1 and IgA2m(2) increased from 61.1 ± 0.9% to 90.0 ± 0.4 and 43.5 ± 2.7% to 87 ± 2.2%, respectively (Figure 5B). However, the almost complete *N*-glycosylation in the tailpiece did not fully compensate for the reduced dimeric IgA formation compared to HEK293F-produced IgA variants. The LmSTT3D co-expression led to an 17 ± 2.7% increase of dimeric to monomeric IgA for IgA1 and an increase of 22.7 ± 6.4% for IgA2m(2) (Figure 5C). The assembly was also not further improved when, MZB1 and LmSTT3D were co-expressed together with IgA variants (Figure S7) suggesting the involvement of additional factors for dimeric IgA formation that are missing in plants.

## Discussion

In this study we examined factors that affect the dimeric IgA formation in *N. benthamiana* which is currently one of the most widely used plant for recombinant protein expression and glyco-engineering (Bally et al., 2018; Montero-Morales and Steinkellner, 2018). IgAs are heavily glycosylated and distinct glycoforms contribute to the overall thermal stability of IgAs (Göritzer et al., 2017), the *in vivo* half-life (Rouwendal et al., 2016) and effector functions (Steffen et al., 2020). Moreover, not only is the *N*- as well as *O*-glycan composition different between plant- and mammalian cell-derived IgAs, but also the degree of glycosylation in the single tailpiece *N*-glycosylation site (Göritzer et al., 2017; Castilho et al., 2018). This underglycosylation at the C-terminal end of the α-HC may be caused by unknown differences in the oligosaccharyltransferase function between mammals and plants (Strasser, 2016; Shrimal and Gilmore, 2019). Mammals contain two distinct OST complexes and the STT3B complex post-translationally glycosylates acceptor sites at extreme C-terminal regions (Ruiz-Canada et al., 2009; Shrimal et al., 2013). Plants have also two distinct STT3 subunits, but their role in co- and post-translational *N*-glycosylation is unknown (Koiwa et al., 2003). Previously it was suggested that partial *N*-glycosylation of the α-HC tailpiece and/or the JC impairs secretory IgA assembly in plants (Westerhof et al., 2015). Our data demonstrate that the *N*-glycosylation in the tailpiece is critical for the formation of dimeric IgAs. Hence, engineering of the plant oligosaccharyltransferase complex toward a more mammalian-like function is a strategy to improve the production of dimeric IgAs. While the site-specific *N*-glycosylation of plant-produced α-HC and the secretory component were shown previously (Dicker et al., 2016; Göritzer et al., 2017), we could determine for the first time the *N*-glycan profile of a plant-produced JC incorporated into dimeric IgA. In contrast to the α-HC tailpiece, an almost complete occupancy was found at the single JC site suggesting that the JC *N*-glycosylation does not contribute to the reduced amounts of dimeric IgAs in plants.

Previously it was revealed that the JC incorporation is the limiting factor for secretory IgA formation (Westerhof et al., 2015). This is consistent with our findings, where we did not observe an increase of dimeric IgA when the amount of infiltrated JC was varied. Along with *N*-glycosylation, our data indicate that there are other factors contributing to the IgA dimerization. When we tested the effect of plant and mammalian ER-resident proteins involved in protein folding and assembly, we did not observe improved dimeric IgA formation. However, the overall yield of the produced IgAs was increased. This finding suggests that the plant proteins BiP, CNX, or CRT are not specifically involved in dimeric IgA formation, but in the assembly or stabilization of the IgA α-HC and κ-LC resulting in higher amounts of monomeric and dimeric IgA. Human ERp44 expression clearly increased the yield, but had no effect on the IgA polymerization suggesting that it is not required for this process in plants. Despite being expressed and correctly retained in the ER in plants, tagged human MZB1 neither affected the yield nor improved the dimeric IgA formation. The precise mechanism for the MZB1-mediated incorporation of the JC into dimeric IgA is currently unknown (Xiong et al., 2019). There is no homolog of MZB1 present in plants and it is possible that the protein is not functional in *N. benthamiana* because a potential interaction partner is missing. MZB1 is a member of a large ER-chaperone complex that includes BiP as well as other folding assistants (Shimizu et al., 2009). Expression of MZB1 variants or the combination of MZB1 with ER-resident mammalian chaperones could be tested in the future to increase the assembly and JC incorporation. In this regard, the use of multi-cassette vectors carrying the α-HC, k-LC, JC as well as potential chaperones on the same vector could overcome unbalanced expression in cells (Westerhof et al., 2015) and further boost the dimeric IgA formation.

In previous studies it was discovered that plant-produced IgAs are poorly secreted and the tailpiece harbors a sequence motif for sorting to the vacuole (Hadlington et al., 2003; Paul et al., 2014; Westerhof et al., 2014). We analyzed the amount of secreted IgAs in the presence or absence of co-expressed JC and could not detect an appreciable difference in secretion (Figure S8). The majority of monomeric and dimeric IgA remained inside the cells, while the IgA in the apoplast was degraded. The absence of major degradation products for the IgA variants inside the cells suggests that they are not targeted to the vacuole. Further studies are required to unravel the subcellular compartment where the majority of the monomeric and dimeric IgAs accumulate.

We were interested to examine whether the *N*-glycan composition is different for monomeric or dimeric IgA variants as reported for monomeric and polymeric serum IgA (Oortwijn et al., 2006). On several *N*-glycosylation sites we observed distinct changes in the *N*-glycan composition with a decrease of truncated *N*-glycans on plant-produced dimeric IgAs. These structures are likely generated in a post-Golgi compartment by β-hexosaminidases (Shin et al., 2017) and differences between monomeric and dimeric IgAs can be explained by changes in the accessibility of the *N*-glycans due to the dimer formation and incorporation of the JC. Whether these changes in *N*-glycan composition cause differences in binding to specific receptors needs to be tested in the future. Previously, we have shown that monomeric IgAs with different *N*-glycans display comparable binding affinities to FcαRI (Göritzer et al., 2019). Here, we performed a kinetic analysis of the dimeric IgA variants and observed similar affinities and kinetics for monomeric IgA. The *N*-glycan composition of dimeric IgA likely does not contribute FcαRI binding and the JC incorporation does not cause steric hindrance as shown for the secretory component (Vidarsson et al., 2001). Moreover, the deduced 1:1 binding stoichiometry for dimeric IgA variants to the receptor is consistent with models proposing that one dimeric IgA binds only two FcαRI, although one dimeric IgA has four binding sites (Bonner et al., 2008; Breedveld and van Egmond, 2019).

In conclusion, we show that functional dimeric IgA binding to the antigen as well as to the most relevant IgA receptor can be produced by transient expression in *N. benthamiana*. The inefficient formation of dimeric IgAs is partly caused by underglycosylation of the *N*-glycosylation in the tailpiece as well as by other factors that need to be uncovered to make plants a suitable expression system for this important class of monoclonal antibodies.

## Data Availability Statement

All datasets generated for this study are included in the article/Supplementary Material.

## Author Contributions

KG, IG, SD, and DM conducted the experiments. KG, IG, DM, and RS analyzed the results. KG, FA, CO, and RS conceived and supervised the experiments. KG and RS wrote the paper. All authors have made a substantial and intellectual contribution to the work and approved it for publication.

## Conflict of Interest

The authors declare that the research was conducted in the absence of any commercial or financial relationships that could be construed as a potential conflict of interest.
