# Supplementary material for: Efficient N-Glycosylation of the Heavy Chain Tailpiece Promotes the Formation of Plant-Produced Dimeric IgA
Source: Front Chem. 2020 Apr 22;8:346. doi: 10.3389/fchem.2020.00346 (PMC7212365; doi:10.3389/fchem.2020.00346)
Supplement: Supplementary file 1 [file Data_Sheet_1.PDF]

# Supplementary Information

# Efficient *N*-glycosylation of the heavy chain tailpiece promotes the formation of plant-produced dimeric IgA

Kathrin Göritzer, Iris Goet, Stella Duric, Daniel Maresch, Friedrich Altmann, Christian Obinger, Richard Strasser

Contents

Figure S1. Relative amounts of monomeric, dimeric and polymeric IgAs.

Figure S2. *N*-glycan analysis of purified monomeric and dimeric IgA1.

Figure S3. *N*-glycan analysis of purified monomeric and dimeric IgA2m(2).

Figure S4. *O*-glycosylation profiles of plant- and HEK293F-derived monomeric and dimeric IgA1.

Figure S5. *N*-glycan analysis of the joining chain (JC).

Figure S6. Co-infiltration of IgAs with different ER-resident proteins in *N. benthamiana* ΔXT/FT.

Figure S7. Co-infiltration of dimeric IgA1 and IgA2m(2) with MZB1 and LmSTT3D.

Figure S8. Secretion and proteolytic degradation of plant-produced IgA1 and IgA2m(2).

Table S1. Kinetic parameters of monomeric (m) and dimeric (d) IgA variant interaction with FcαRI.

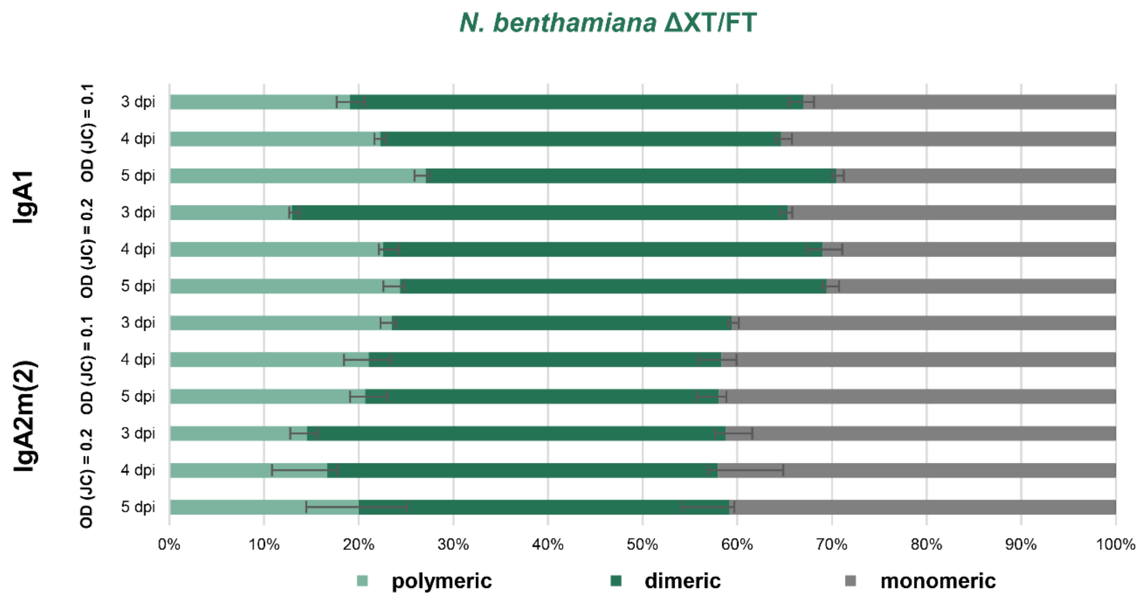


**Figure S1. Relative amounts of monomeric, dimeric and polymeric IgAs.** The relative amounts of monomeric, dimeric and polymeric IgA species produced in *N. benthamiana* ΔXT/FT were determined as the mean ± standard deviation from three independent SE-HPLC chromatograms using peak integration.

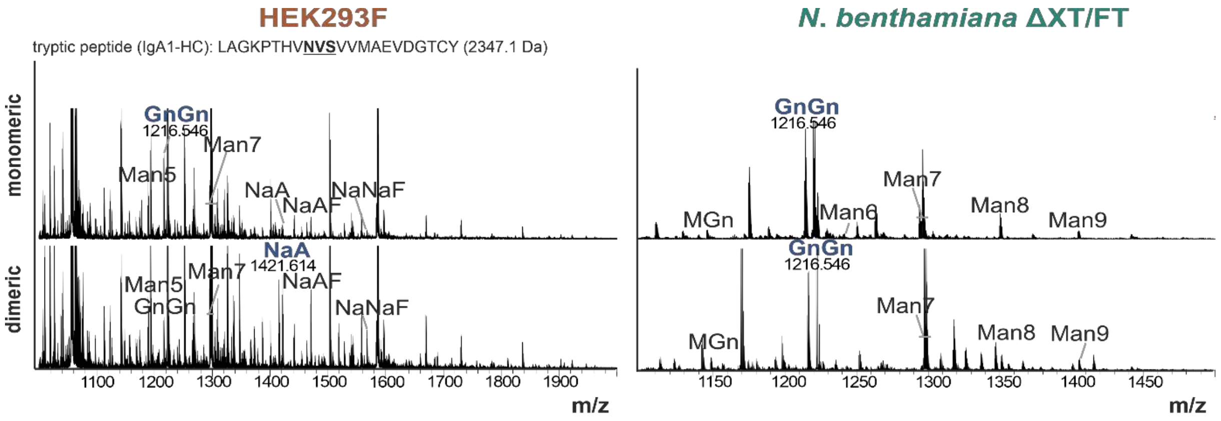


**Figure S2. *N*-glycan analysis of purified monomeric and dimeric IgA1.** Representative MS-spectra ($[M+3H]^{3+}$) of the tryptic glycopeptide containing the tailpiece resident NVS glycosylation site of the α-HC of HEK293F- and plant-produced IgA1. The most abundant glycoform is highlighted in blue. Please note that highly branched complex *N*-glycan structures are likely underrepresented in the HEK293F-derived spectra due to inconsistent elution during LC-ESI-MS.

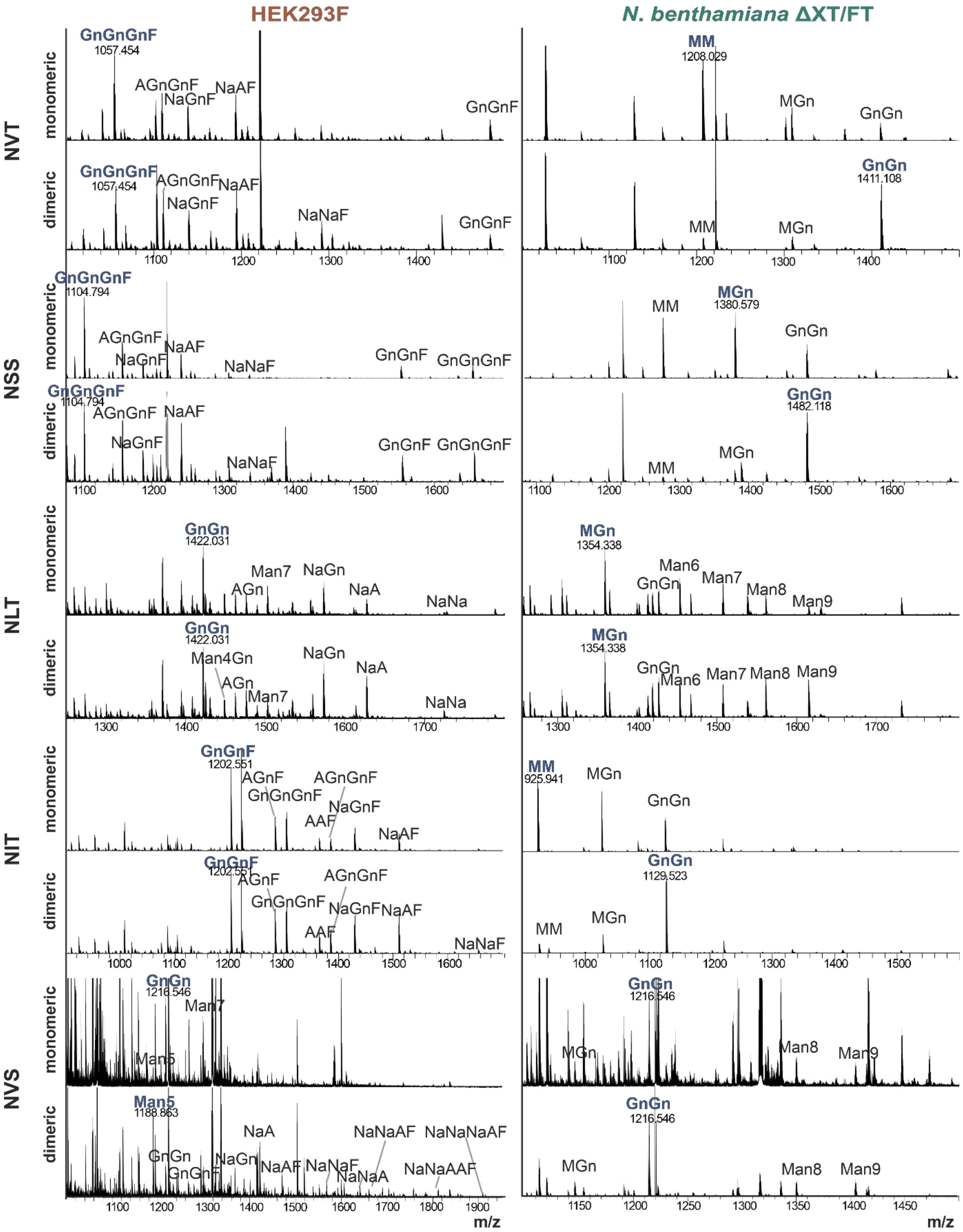


**Figure S3.** ***N*****-glycan analysis of purified monomeric and dimeric IgA2m(2).** MS-spectra of the tryptic glycopeptides "SVTWSESGQNVTAR", "HYTNSSQDVTVPCR", "LSLHRPALEDLLLGSEANLTCTLTGLR", "TPLTANITK" and "LAGKPTHVNVSVVMAEVDGTCY" derived from the α-HC of purified HEK293F- and plant-derived monomeric and dimeric IgA2m(2). In each spectrum, the most abundant glycoform is highlighted in blue.

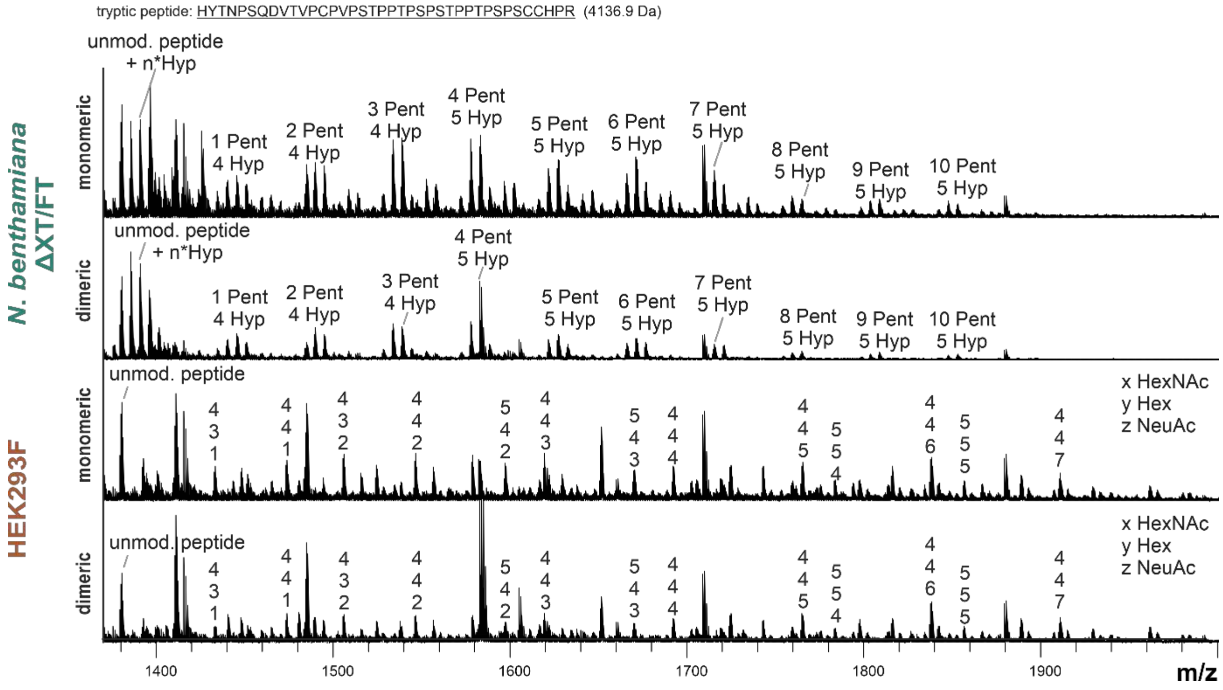


**Figure S4. *O*-glycosylation profiles of plant- and HEK293F-derived monomeric and dimeric IgA1.** Mass spectra of the hinge-region peptides of plant-produced ($[M+3H]^{3+}$) and HEK293F-produced ($[M+3H]^{3+}$ and $[M+4H]^{4+}$) IgA1 are shown. Glycosylated peaks are indicated as follows: pentoses (Pent), hydroxyproline (Hyp), putative *N*-acetylgalactosamine (HexNAc), hexoses (Hex) and sialic acid (NeuAc).

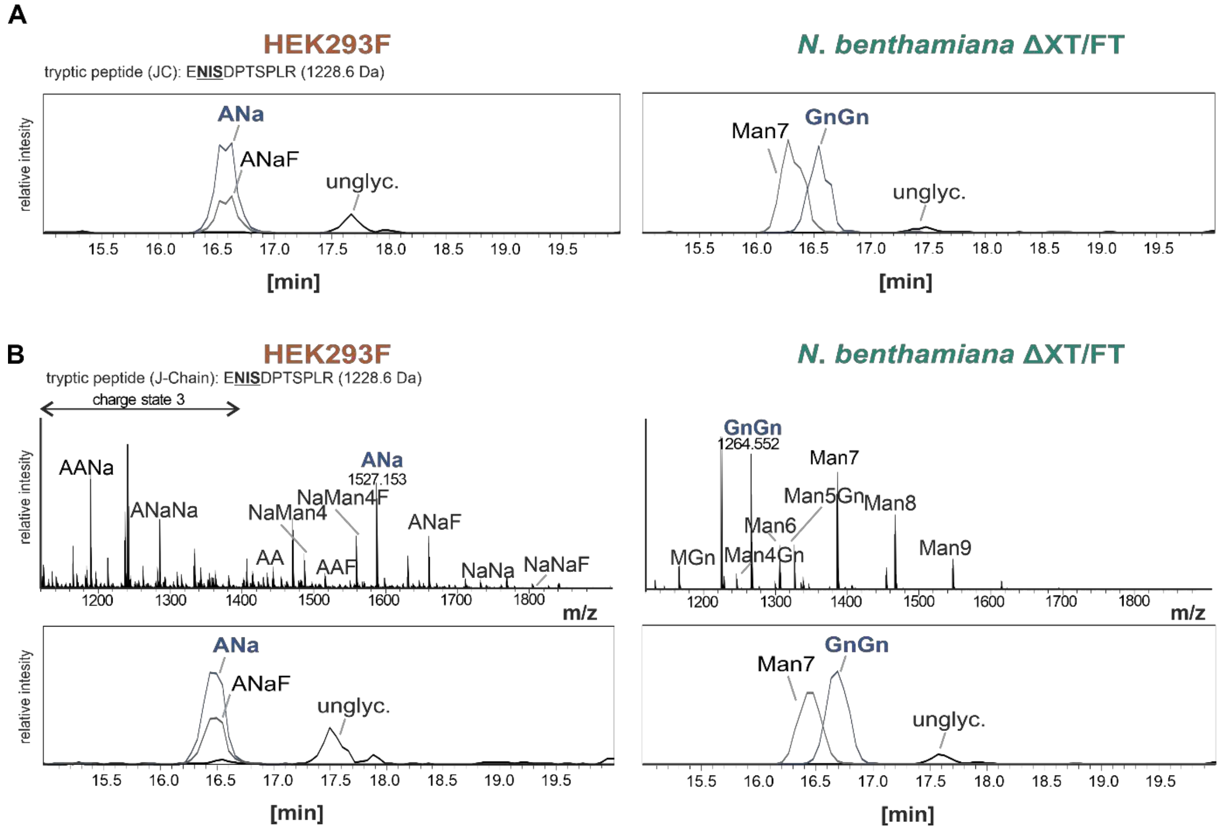


**Figure S5.** ***N*-glycan analysis of the joining chain (JC). (A)** Overlay of the relative LC-ESI-MS chromatograms of the tryptic glycopeptides containing the single NIS glycosylation site of the joining chain (JC) of HEK293F- and plant-produced dimeric IgA1. Chromatograms of the unglycosylated (unglyc.) peptide and peptides carrying the two most abundant glycoforms are shown. *N*-glycans are abbreviated according to the ProGlycAn system (www.proglycan.com). **(B)** MS-spectra ($[M+2H]^{2+}$and $[M+3H]^{3+}$) of the tryptic glycopeptide containing the single NIS glycosylation site of the JC of HEK293F- and plant-produced dimeric IgA2m(2) and overlay of the relative LC-ESI-MS chromatograms of the tryptic glycopeptides without modifications (unglyc.) and with the two most abundant glycoforms. In each spectrum, the most abundant glycoform is highlighted in blue.

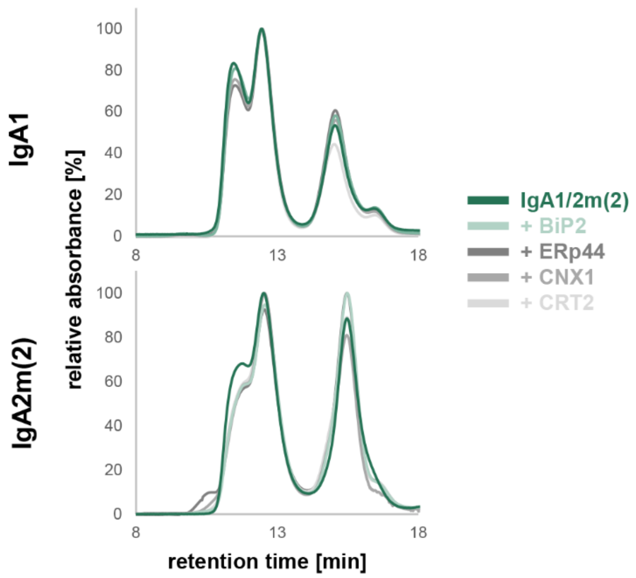


**Figure S6. Co-infiltration of IgAs with different ER-resident proteins in *N. benthamiana* ΔXT/FT.** Overlay of normalized SE-HPLC chromatograms of affinity-purified dimeric IgA1 and IgA2m(2). One representative out of three independent experiments is shown.

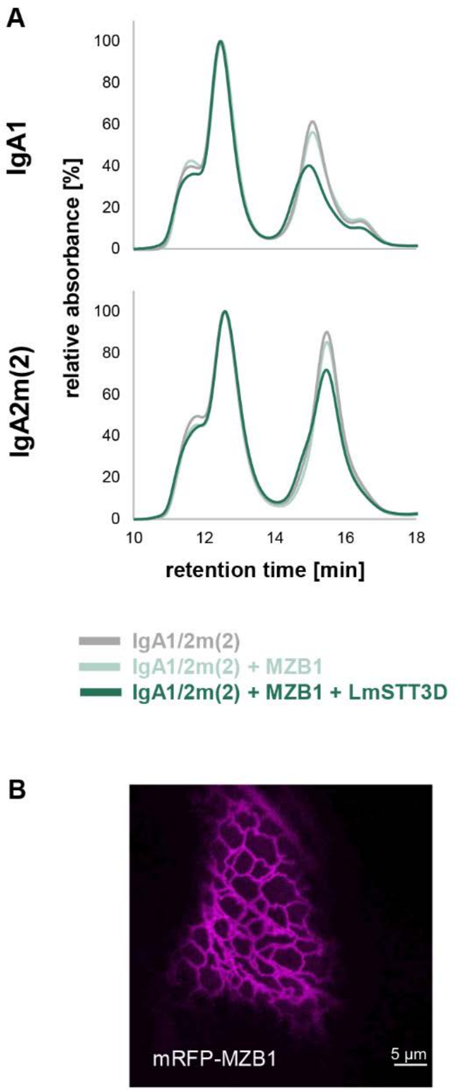


**Figure S7. Co-infiltration of dimeric IgA1 and IgA2m(2) with MZB1 and LmSTT3D. (A)** Overlay of normalized SE-HPLC chromatograms of affinity-purified dimeric IgA1 and IgA2m(2). One representative out of three independent experiments is shown. **(B)** To confirm the ER-localization, mRFP-MZB1 was transiently expressed in *N. benthamiana* leaf epidermal cells and analyzed by confocal microscopy as described previously (Shin *et al.* 2017).

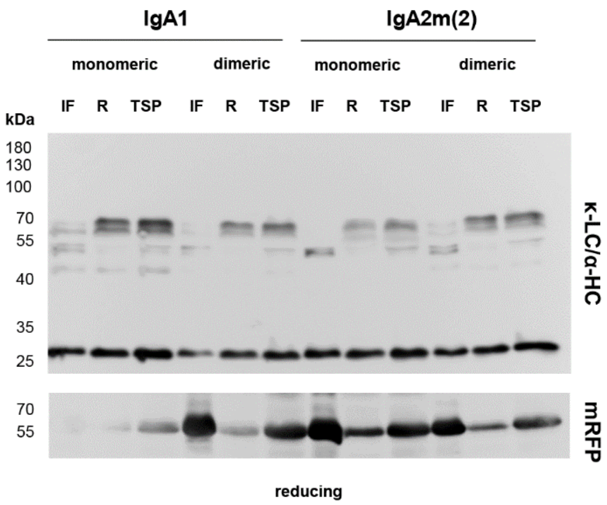


**Figure S8. Secretion and proteolytic degradation of plant-produced IgA1 and IgA2m(2).** Reducing SDS-PAGE and immunoblotting of intercellular fluid (IF), remaining crude extract (R) and total soluble protein (TSP) of infiltrated leaves. Monomeric and dimeric IgA1 and IgA2m(2) were co-infiltrated with a Sec-Fc-mRFP construct that is very efficiently secreted to the apoplast (Shin *et al.* 2017). Blots were either visualized by an anti κ-LC and α-HC antibody (A7164 and A0295 from Sigma) or by an anti-mRFP antibody (RFP antibody [6g6] from Chromotek). One representative result out of two independent experiments is shown.

**Table S1.** Kinetic parameters of monomeric (m) and dimeric (d) IgA variant interaction with FcαRI. Rate constants are an average of three independent SPR experiments at five different concentrations ± standard deviation.

| ligand | analyte | $k_{on}$ [(M s)$^{-1}$] | | | $k_{off}$ [s$^{-1}$] | | | $K_D$ [nM] | | |
|---|---|---|---|---|---|---|---|---|---|---|
| **mIgA1$_{HEK}$** | FcαRI | $4.4 \times 10^5$ | ± | $3.0 \times 10^4$ | 0.050 | ± | 0.002 | 108.96 | ± | 12.42 |
| **mIgA1$_{NB}$** | FcαRI | $5.8 \times 10^5$ | ± | $6.6 \times 10^4$ | 0.061 | ± | 0.009 | 103.45 | ± | 11.57 |
| **dIgA1$_{HEK}$** | FcαRI | $3.6 \times 10^5$ | ± | $5.4 \times 10^3$ | 0.055 | ± | 0.003 | 143.94 | ± | 3.30 |
| **dIgA1$_{NB}$** | FcαRI | $3.7 \times 10^5$ | ± | $3.3 \times 10^4$ | 0.057 | ± | 0.003 | 151.30 | ± | 6.63 |
| **mIgA2m(2)$_{HEK}$** | FcαRI | $3.1 \times 10^5$ | ± | $2.8 \times 10^4$ | 0.050 | ± | 0.003 | 156.64 | ± | 4.91 |
| **mIgA2m(2)$_{NB}$** | FcαRI | $3.4 \times 10^5$ | ± | $2.1 \times 10^3$ | 0.055 | ± | 0.003 | 159.89 | ± | 8.52 |
| **dIgA2m(2)$_{HEK}$** | FcαRI | $2.6 \times 10^5$ | ± | $1.1 \times 10^4$ | 0.049 | ± | 0.003 | 181.64 | ± | 5.77 |
| **dIgA2m(2)$_{NB}$** | FcαRI | $2.7 \times 10^5$ | ± | $1.5 \times 10^4$ | 0.049 | ± | 0.007 | 225.69 | ± | 12.57 |
